# Effect of Superfine Cement Modification on Properties of Coral Aggregate Concrete

**DOI:** 10.3390/ma16031103

**Published:** 2023-01-27

**Authors:** Fei Wang, Jianmin Hua, Xuanyi Xue, Neng Wang, Feidong Yan, Dou Feng

**Affiliations:** 1 School of Civil Engineering, Chongqing University, Chongqing 400045, China; 2 School of Management Science and Real Estate, Chongqing University, Chongqing 400045, China

**Keywords:** coral aggregate concrete, superfine cement, aggregate modification, physical property, mechanical property, concrete workability

## Abstract

In marine engineering, using corals as aggregates to prepare concrete can reduce both the exploitation of stones and the transportation cost of building materials. However, coral aggregates have low strength and high porosity, which may affect the workability and mechanical properties of concrete. Hence, superfine cement is used innovatively in this study to modify coral aggregates; additionally, the effects of the water–cement ratio and curing time on the water absorption and strength of modified coral aggregates are investigated. Modified coral aggregate concrete is prepared, and the effect of using modified superfine cement on its workability and strength is investigated. Experimental results show that when the water-cement ratio exceeds 1.25, the slurry does not form a shell on the surface of the coral aggregates and the water absorption of the coral aggregates increases significantly. The strength of the modified coral aggregates cured for a short duration is slightly lower than that of unmodified coral aggregates, whereas that cured for 28 days is approximately 20% higher than that of unmodified coral aggregates. Using superfine cement to modify coral aggregate concrete can improve its workability, but not its compressive properties.

## 1. Introduction

Owing to the development and increased utilisation of the ocean, a significant amount of marine infrastructure is being constructed, which necessitates more building materials. Corrosion-resistant structural steels have been developed, such as fibre-reinforced polymer bars [1,2,3], stainless-clad bimetallic steel [4,5,6,7,8], and titanium-clad bimetallic steel [9,10,11]. The application of reinforced concrete structures in marine engineering is becoming increasingly extensive. Concrete, as one of the main raw materials for reinforced concrete structures, is in high demand. Aggregates constitute 70% to 80% of concrete volume and contribute significantly to concrete [12,13]. For construction on islands and reefs distant from the mainland, the transportation of sand aggregates significantly increases the construction cost [14]. Therefore, alternative materials that satisfy engineering requirements must be identified. Sea sand has been used as a fine aggregate to prepare concrete [15,16,17]. Coral is a relatively easy building material for marine engineering. In the 1830s, corals were first used as aggregates in concrete. After investigating coral aggregate concrete buildings in Guam, Howdyshell [18] concluded that coral aggregates can be used in concrete structures. In recent years, scholars have extensively investigated coral aggregate concrete. Wu et al. [19] investigated the physical and mechanical properties of different coral aggregates, revealed the pore morphology and distribution of coral aggregates. The results indicated that the mechanical properties of coral aggregate are poor due to its high porosity. Ma et al. [20] prepared high-strength coral aggregate seawater concrete and investigated its impact resistance, which illustrated that the high porosity of coral aggregate led to a large dynamic increase factor of concrete. Chen et al. [21] revealed the internal damage and fracture mechanism of coral aggregate concrete via numerical simulations and discovered that microcracks initiated from concrete pores expanded into aggregates. Cai et al. [22] performed a comparison between coral aggregate concrete and ordinary Portland cement concrete, which revealed the low strength of coral aggregate under impact load. Furthermore, the energy required for coral aggregate concrete to initiate the main crack is much lower than that of ordinary Portland cement concrete. Zhou et al. [23] discovered that coral aggregates had a lower strength but higher porosity than sand gravel, which resulted in the strength of coral aggregate concrete being lower than that of ordinary concrete. The above research indicates that although coral aggregate can be used to prepare concrete, the workability and mechanical property of coral aggregate concrete are relatively poor.

To effectively improve the workability and mechanical properties of coral aggregate concrete, the coral aggregates to be used must be pretreated. Coral aggregates are characterised by low density, high crushing index, high porosity, and high water absorption, which are typical of lightweight aggregates; therefore, coral aggregates can be regarded as a lightweight aggregate. To modify lightweight aggregate concrete, researchers typically use inorganic cementitious materials to treat the aggregates, reduce the aggregate porosity, and improve the aggregate strength and durability. He et al. [24] formed a slurry comprising 80% cement, 10% fly ash, and 10% silica fume to modify recycled aggregates of broken brick concrete. The results showed that the slurry increased the apparent density and strength of recycled brick concrete aggregates. Yang et al. [25] investigated the strengthening effect of cement paste on the mechanical properties of broken brick aggregate concrete. It was found that the broken brick aggregate concrete demonstrated better mechanical properties and higher resistance to chloride ion migration after being treated with a cement-coal fly ash slurry via soaking for 4 h. Compared with before modification, the compressive strength of the brick aggregate concrete increased by 22–34% after modification. Hossein et al. [26] improved the durability of recycled concrete aggregate concrete by soaking it in silica fume paste. The results showed that the water absorption of recycled aggregates decreased by 14–22%, and the resistivity increased significantly after modification with silica fume paste. The above research shows that the inorganic cementitious materials modification method can improve the mechanical properties and durability of lightweight aggregate concrete. However, the above modification methods still cannot satisfy the requirements of coral aggregate concrete, due to the high porosity and low strength of coral aggregate. At present, the research on modification methods of coral aggregate is relatively limited, and the influence of modification on aggregate properties and concrete performance is still unclear.

Considering the high porosity of coral aggregates and the typical methods used to modify porous lightweight aggregates, superfine cement is used innovatively in this study to modify coral aggregates. Changes in the water absorption and strength of coral aggregates before and after modification are investigated. Modified and unmodified coral aggregates are used to prepare coral aggregate concrete. Subsequently, the effects of using modified superfine cement on the workability, strength, and failure mode of coral aggregate concrete are revealed. The aim of this study is to provide a new method for modifying coral aggregates, which can promote the use of coral aggregate in the construction of islands and reefs. The experimental results are conducive to the design and construction of modified coral aggregate concrete structures.

## 2. Materials and Methods

### 2.1. Materials

#### 2.1.1. Coral

**Figure 1:**
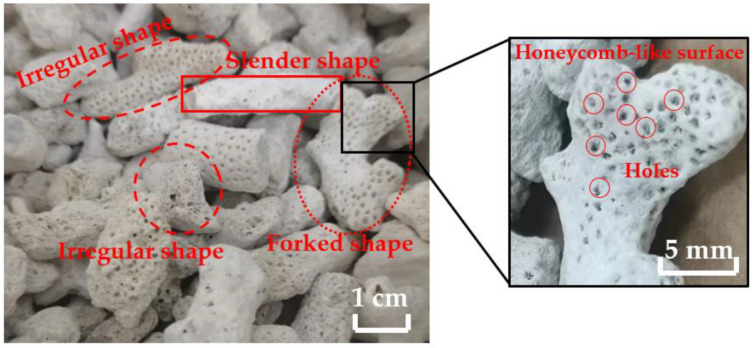
Coral.

**Figure 2:**
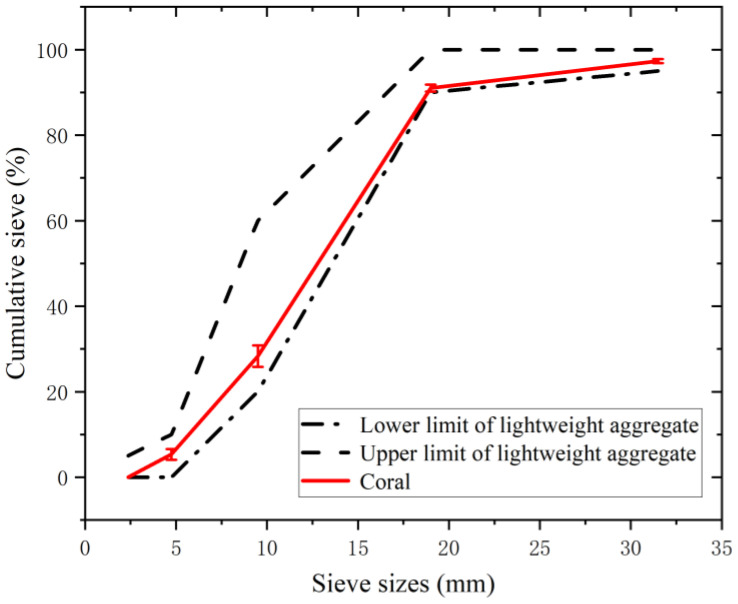
Coral aggregate gradation.

The coral aggregates used in this study were obtained from the South China Sea. As shown in Figure 1, corals have different shapes, which may be elongated, forked or irregular. In addition, there are many holes in the coral, showing a honeycomb-like surface. The aggregate particle size distribution indicates the proportion of aggregates with different particle size ranges relative to the total aggregate quality. The particle size distribution affects the slump, expansion, consistency, bleeding rate, and other performances of concrete mixtures [27,28], as well as the mechanical properties [29] and durability [30] of concrete. In accordance with GB/T 17431.1-2010 [31], the coarse aggregates used in this experiment were screened and analysed to obtain the gradation of the coral aggregates, as shown in Figure 2. The experimental coral aggregates satisfied the requirements of GB/T 17431.1-2010 [31] in terms of the upper and lower limits of gradation for lightweight aggregates measuring 5–20 mm. Table 1 lists the physical properties of the coral aggregates used in the experiment.

#### 2.1.2. Cement and Superfine Cement

Owing to the high porosity of coral aggregates, the coral aggregates were modified by soaking them in a high-activity micro-inorganic cementitious material. The internal pores and microcracks of the coral aggregates were filled with slurry to improve the internal structure of the aggregates as well as the workability and mechanical properties of the concrete. Ordinary Portland cement, which is typically used for modification, features a large particle size; thus, the particles do not permeate easily into microcracks. Generally, particles of ordinary Portland cement can only penetrate into pores larger than 0.2 mm; therefore, the modification effect on porous aggregates is unsatisfactory, and improvements to fine pores require cement with a smaller particle size. Superfine cement is a high-performance cement-based grouting material with a much higher fineness than ordinary cement [32]. It has higher strength and better groutability. Because of the superfine grain size and larger surface area of cements, superfine cement slurry has good fluidity and particle filling [33]. Therefore, to fill the fine pores of coral aggregates and reduce the water absorption and crushing index of aggregates, superfine cement was adopted to strengthen and modify the coral aggregates. The superfine cement used in the current experiment was 1250 mesh superfine cement produced by China Resources, and its performance index is shown in Table 2. The cement used to prepare concrete was P.O 42.5 ordinary Portland cement produced by Sichuan Esheng Cement Plant, which satisfies the requirements of GB175-2020 [34], and its performance index is shown in Table 3.

#### 2.1.3. Seawater

Water is a basic component of concrete that also significantly affects its durability, strength, and workability [35,36,37]. Using seawater to prepare concrete can solve the problem of freshwater resource shortage on islands. However, seawater contains numerous ions, which may affect the performance of concrete. Previous studies have shown that Cl^-^ in seawater affects the strengthening of concrete [38] and that SO_4_^2−^ may result in the expansion and cracking of concrete by affecting the formation time of gypsum and ettringite [39]. In the current experiment, artificial seawater was used to mix concrete and prepare a slurry of modified coral aggregates. The artificial seawater was prepared based on ASTM D1141-2013 [40]. The composition of the seawater is shown in Table 4. Similar configuration methods have been used in several studies [41,42,43].

#### 2.1.4. Sea Sand

The mineral composition of sea sand is similar to that of river sand; however, the content of shells and organic matter in sea sand deteriorates the strength and durability of concrete [44]. In addition, sea sand contains more ions than river sand. In this study, undiluted sea sand was obtained from the South China Sea. Based on the classification method presented in GB/T 14684-2011 [45], the grain size distribution of sea sand belongs to Zone II, and the fineness modulus is between 2.3 and 2.6. The particle size distributions are listed in Table 5. The mud, mica, and shell contents of sea sand were all less than 1.0%, 1.0%, and 5.0%, respectively.

### 2.2. Coral Aggregate Modification

The method of preparing aggregates reinforced by modified slurry is to soak aggregates in the slurry for a period of time, remove the aggregates before the initial setting of the slurry, and perform natural curing after the surface slurry has solidified. This process ensures a tight slurry shell on the aggregate surface. However, it is disadvantageous to the aggregates in two aspects: the smooth shell on the aggregate surface reduces the friction between the aggregates and cement gels as well as reduces the bite force between the aggregates, which improves the mechanical properties of the concrete; additionally, the slurry shell forms additional pores, which increases the water absorption of the aggregates, thereby improving the water absorption of the modified aggregates to an extent greater than that before modification. The appropriate slurry water-cement ratio (W/C) and modification process must be identified. When the W/C of the slurry is relatively large, the slurry will be very thin, making it impossible to fill the aggregate pores when the slurry is solidified. On the contrary, the slurry will be very thick when the W/C is relatively small. The thick slurry will form a thick slurry shell outside the coral aggregate. The thick shell may affect the strength of the aggregate and lead to unexpected water absorption. Based on the results of pre-test, the values of W/C of slurry was determined as 1.0, 1.25, 1.5 and 2.0.

The superfine cement was placed in a stirrer, and artificial seawater was added to the stirrer based on the W/C values specified. Both materials were mixed well. After the slurry was prepared, coral aggregates were soaked in it and stirred to ensure that the aggregates were in full contact with the slurry. When the aggregates were soaked, they were stirred once every hour to avoid bonding between the aggregates and ensure the uniformity of the modification. Before the initial set was formed, the aggregates were removed from the slurry. The aggregates were continuously sieved using a dense mesh to filter out the excess modified slurry and avoid the formation of an excessively thick slurry shell on the aggregate surface. Subsequently, the aggregates were arranged flat on a net and dried at room temperature for 24 h, during which the aggregates were flipped to prevent them from bonding with each other. In order to reduce the impact of the environment [46,47], coral aggregates were placed in the standard environment (20 ± 2 °C and 95% relative humidity) for curing for 3 d, 7 d, 14 d and 28 d, respectively.

### 2.3. Physical and Mechanical Property Test

To investigate the effect of superfine cement modification on the coral aggregate concrete, the properties of coral aggregate concrete and coral aggregates before and after the modification were evaluated. When mixing concrete, the dry aggregate will absorb the moisture of the concrete mixture, resulting in a change in the effective W/C of concrete, thus affecting the construction process and mechanical properties of concrete. Therefore, the water absorption of aggregate must be considered when designing the concrete mix proportion. Furthermore, coral aggregate has a high porosity, which may cause its water absorption to be different from that of ordinary aggregate. Therefore, it is necessary to clarify its water absorption. In accordance with the GB/T 17431.2-2010 standard [48], dry coral aggregates were sieved and soaked; subsequently, their masses before and after water absorption were weighed. The water absorption rate of the coral aggregates was calculated using Equation (1) [48], where *m_0_* is the mass of the aggregates in the saturated surface condition, and m_1_ is the mass of the dry aggregates. Concrete is composed of water, cement and aggregate, constituting 70% to 80% of concrete volume. Aggregate strength greatly affects concrete strength. Therefore, it is necessary to clarify the strength of the aggregate. The strength of the coral aggregates was evaluated using the crushing index (*C_r_*). The mass of the coral aggregates (*G_0_*) was weighed, and then loaded using a pressure tester. Subsequently, the assembly was pressurised to 200 kN at a rate of 1 kN/s and then unloaded. A 2.5 mm sieve was used to remove crushed fine particles, and the mass remaining on the sieve (*G_1_*) was weighed. The *C_r_* of the coral aggregates was calculated using Equation (2) [49].

The concrete shall meet the strength requirements to ensure the safety of the structure. In addition, it shall have good workability to facilitate transportation and construction. The physical and mechanical properties of the coral aggregates differed significantly from those of natural aggregates. Therefore, the workability and mechanical properties of seawater and sea sand concrete with coral aggregates differed significantly from those of seawater and sea sand concrete with natural aggregates. To investigate the effect of superfine cement modification on the coral aggregate concrete, modified coral aggregate concrete was prepared by replacing natural coarse aggregates with coral aggregates at replacement rates of 0%, 25%, 50%, 75%, and 100% by volume. There were three specimens in each aggregate replacement rate. The effects of the modification and replacement rate on the workability of coral aggregate seawater sea sand concrete were analysed. The mix ratio of the concrete is shown in Table 6. To reflect the workability of the concrete, the slump, expansion, and bleeding rate of the newly mixed modified coral aggregate concrete were tested based on GB/T 50080-2016 [50]. Axial compression experiments were performed to evaluate the mechanical properties of the concrete. Specimens measuring 100 mm × 100 mm × 300 mm were prepared by mixing coral aggregate concrete. The specimens were subjected to standard maintenance for 28 d, after which their compressive performance was evaluated using a pressure-testing machine. The loading rate was maintained at 0.5 MPa/s until the specimen was destroyed. The deformation of the specimen during compression was recorded using a displacement meter. The experimental procedure satisfied the requirements of GB/T 50081-2019 [51].

(1) $$\rho =\frac{m_{0}-m_{1}}{m_{1}}\times 100\%$$

(2) $$C_{r}=\frac{G_{0}-G_{1}}{G_{0}}\times 100\%$$

## 3. Results and Discussion

### 3.1. Water Absorption of Coral Aggregate

**Figure 3:**
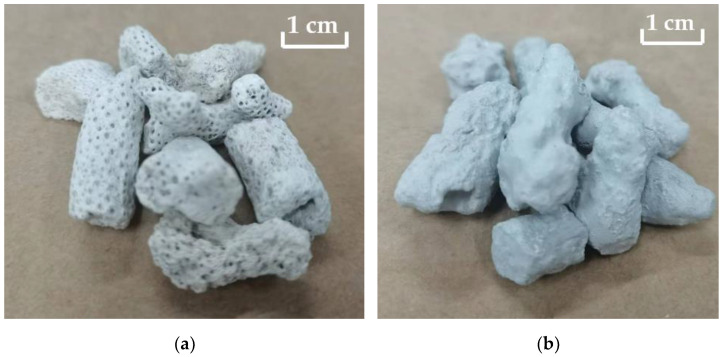
Appearance of coral aggregates: (**a**) unmodified; (**b**) modified.

**Figure 4:**
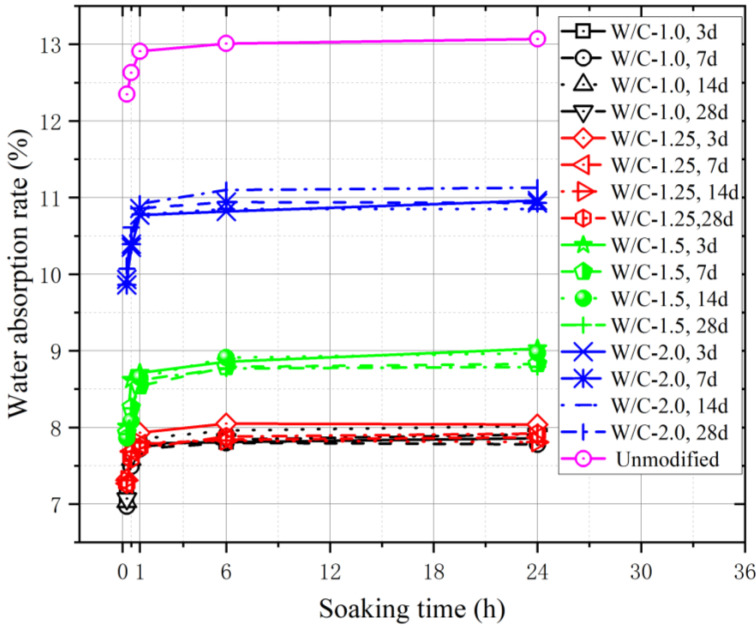
Water absorption of coral aggregates after different soak times.

**Figure 5:**
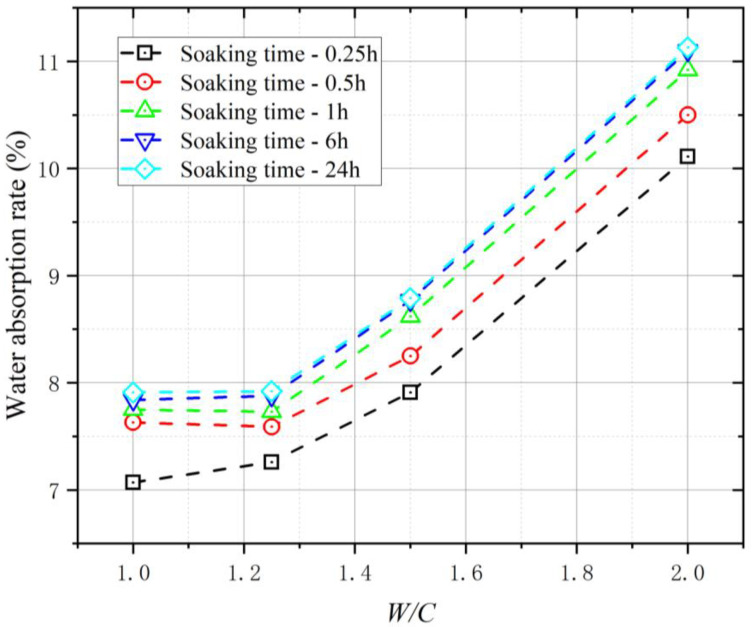
Effect of W/C of cement on water absorption of coral aggregates.

**Figure 6:**
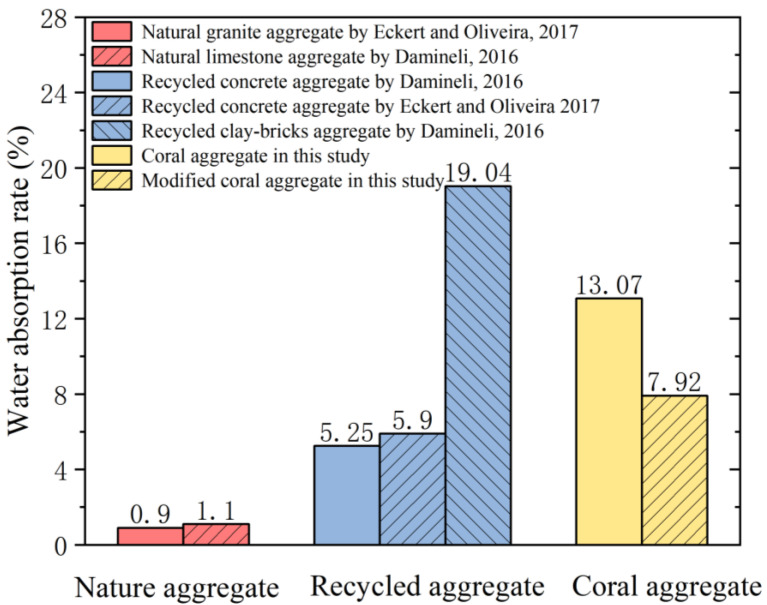
Comparison of water absorption of different types of aggregates [54,55].

When preparing concrete, the water content of the aggregate affects the water and aggregate consumption of concrete. To ensure the workability and strength of concrete, the water absorption of the aggregate should be considered when designing the mix proportion. The surface of the modified coral aggregates (Figure 3) formed a layer of slurry shell, which appeared greyish-white. After modification, the aggregate became smoother and the number of pores reduced significantly, which considerably affected the water absorption of the coral aggregates. The water absorption of the coral aggregate was measured at 0.15, 0.5, 1, 6, and 24 h of immersion to understand its variation with immersion time, as shown in Figure 4. Compared with unmodified coral aggregates, the coral aggregates modified by superfine cement paste exhibited a significantly reduced level of water absorption after immersion. When W/C was 1.0, the water absorption of the modified coral aggregate was only 60% of that of the unmodified coral aggregate. Within 1 h of immersion, the water absorption capacity of the aggregates increased significantly. The water absorption of the aggregate reached 97% to 99% of the 24 h water absorption after soaking for 1 h. Therefore, the coral aggregates can be assumed to have reached the saturated-surface-dry state after 1 h of immersion. Thus, the coral aggregates should be soaked for more than 1 h in advance when used for fabricating coral aggregate concrete. As shown in Figure 4, the effect of the curing age of the modified coral aggregates on the water absorption is negligible. This is because the final setting time of the aggregate surface slurry is less than 3 d, and the slurry shell has already solidified during the curing time test on the third day, which effectively inhibited the water absorption and discharge effect of the aggregate. Therefore, the water absorption rate of the aggregate under different curing times was considerably lower than that before modification; however, the former did not differ significantly. Meanwhile, the W/C significantly affected the water absorption. Figure 5 shows the water absorption of the coral aggregates modified by cement slurry of different W/C values after 28 d of curing. The water absorption rate of the aggregate increased gradually with the W/C of the slurry. When the W/C increased from 1.0 to 1.25, the change range of aggregate water absorption was less than 2%. After the W/C exceeded 1.25, the water absorption of the aggregate increased significantly. When the W/C was 1.5, the water absorption increased by 11.13%. When the W/C was 2.0, the water absorption is 40.71% higher than when W/C = 1.0. This is because when the W/C was less than 1.25, although a denser slurry shell was formed on the surface of the aggregate, an overly thick shell formed additional pores, thus resulting in an insignificant decrease or a slight increase in the water absorption of the aggregate. When the W/C exceeded 1.25, the slurry was too thin to form a slurry shell on the surface of the coral aggregates, which resulted in an unsatisfactory filling and wrapping effect on aggregate pores and a significant increase in water absorption. Numerical fitting can show the evolution law of data and is often performed for data analysis [52,53]. Equation (3) was proposed to quantify the relationship between the W/C and the water absorption of modified coral aggregates via numerical fitting, where ρ represents the water absorption rate of the modified coral aggregates and ω represents the W/C of the slurry. The water absorption of different types of aggregates was compared (Figure 6). It can be seen that the water absorption of modified coral aggregate was bigger than that of recycled concrete aggregate, but less than that of recycled clay bricks aggregate.

(3) $$\rho =2.44\times \omega^{2}-4.37\times \omega +8.96$$

### 3.2. Strength of Coral Aggregates

**Figure 7:**
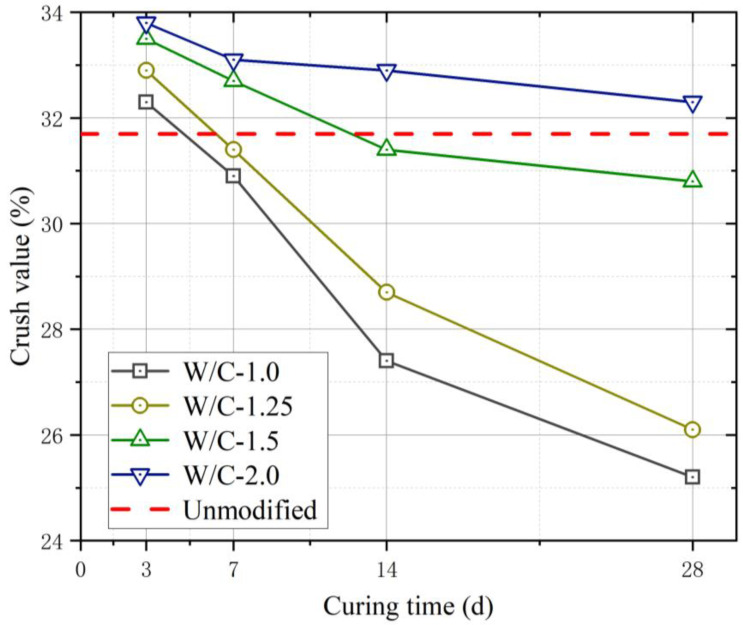
C_r_ of coral aggregates modified by slurry of different W/C values.

**Figure 8:**
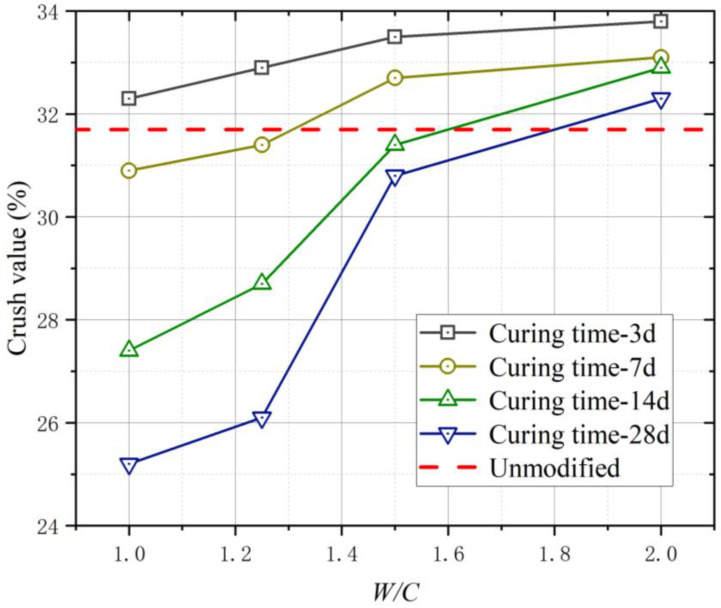
C_r_ of modified coral aggregates with different curing times.

**Figure 9:**
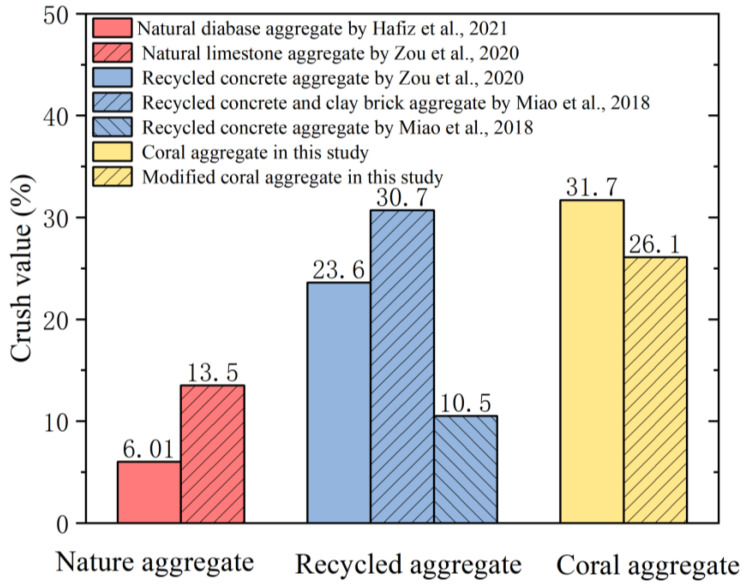
Comparison of C_r_ of different types of aggregates [56,57,58].

Tests were conducted to determine the effect of strength modification on the coral aggregates. Based on Figure 7, the crush value (C_r_) of the aggregates at each W/C was greater than that before modification for curing times of 3 and 7 d. This is owing to the insufficient strength enhancement of the cement slurry encapsulating the aggregates in a short duration. The cement slurry was susceptible to breakage under the pressure of the testing machine and fine particles were filtered using a 2.5 mm sieve, which resulted in an increase in C_r_. Figure 8 shows the C_r_ of the modified coral aggregates with different curing times. When the W/C of the slurry was 1.0 and 1.25, a dense slurry protective layer was formed on the surface of the aggregate; thus, the aggregate strength increased significantly after 14 and 28 d of curing. After 28 days of curing, the C_r_ of the aggregates with W/C of 1.0 was decreased by 21.98%. However, when the W/C was 1.5 and 2.0, the C_r_ of the coral aggregates decreased slightly with the increase of curing time. the C_r_ variation range of aggregate with W/C of 2.0 was within 4.43%. This may be due to the extremely high W/C of the superfine cement slurry and the inadequate slurry attached to and absorbed into the aggregate. In addition, a slurry with a high W/C cannot solidify for a long time, thus causing the slurry to be lost easily during the curing process; consequently, the formation of a compact slurry protective layer on the surface of the aggregate is hindered. Owing to these reasons, the C_r_ of the aggregates did not differ significantly from that before modification even at curing age of 14 and 28 d. The experimental results suggest that the slurry W/C should not be less than 1.25. Additionally, the modified aggregate must be maintained for 28 d, and until the strength of the slurry on the aggregate surface develops to a certain extent, it cannot be used for pouring concrete. The C_r_ of different types of aggregates was compared (Figure 9). It can be seen that the C_r_ of modified coral aggregate was between recycled concrete aggregate and recycled concrete and clay brick aggregate.

### 3.3. Workability of Concrete

**Figure 10:**
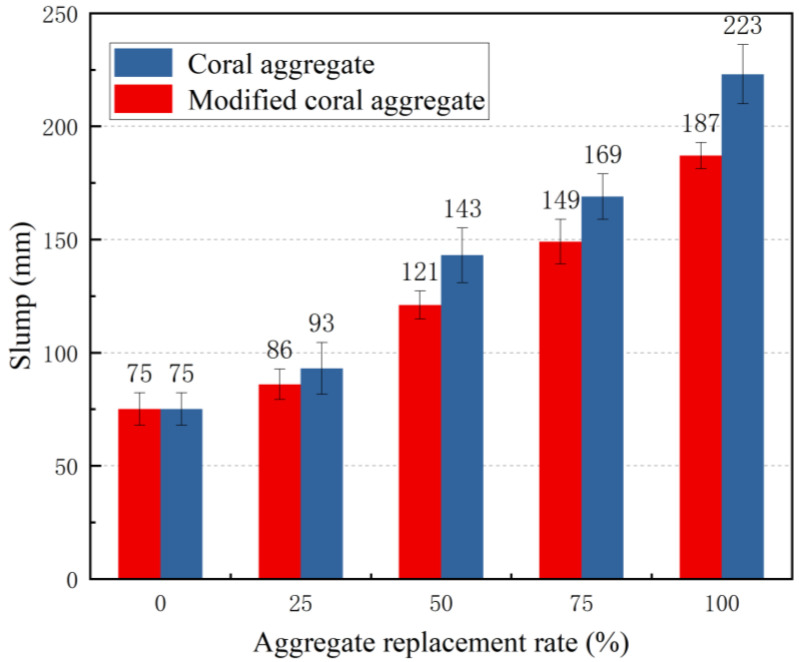
Relationship between aggregate replacement rate and slump.

**Figure 11:**
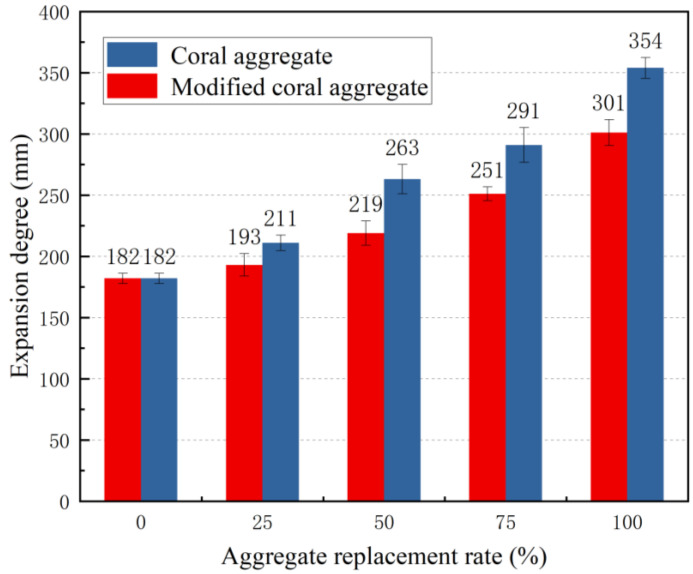
Relationship between aggregate replacement rate and expansion degree.

Based on the discussion presented in Section 3.1 and Section 3.2, concrete was fabricated using coral aggregates modified by a slurry with a W/C of 1.25 and cured for 28 d. The natural coarse aggregates were replaced by volume at 0%, 25%, 50%, 75%, and 100% replacement rates to analyse the effects of modification and replacement rate on the workability of seawater and sea sand concrete. The slump and expansion of concrete, which reflect the fluidity of concrete as well as the cohesion and water retention properties of concrete, are important indicators for determining the workability of concrete. Figure 10 and Figure 11 illustrate the effects of the modification and replacement rate on the slump and expansion of seawater sea sand concrete. As the coral aggregate replacement rate increased, the slump and expansion increased; meanwhile, the slump and expansion of the modified coral concrete at the same replacement rate were significantly lower than those before modification. When the replacement rate was 100%, the slump and expansion of the modified concrete reduced by 16.14% and 14.97%, respectively. With the same concrete mix proportion, the slump of modified coral aggregate concrete was 2.97 times that of gravel aggregate concrete. This was primarily attributed to the significant water absorption and discharge effects of the coral aggregates. During mixing, the pre-wetted aggregate released water into the concrete mixture, thus increasing the concrete slump and expansion. After aggregate modification, the water absorption and discharge effects of the concrete improved; consequently, the slump and expansion of the concrete decreased and the cohesiveness and water retention properties of the concrete improved.

**Figure 12:**
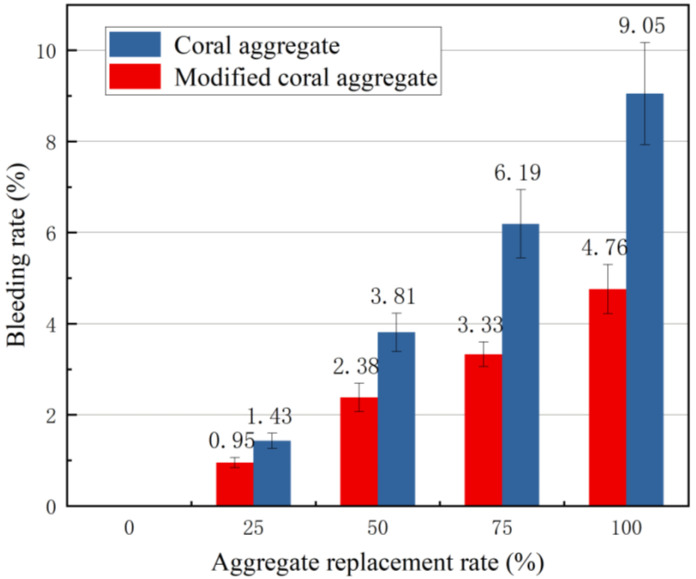
Relationship between aggregate replacement rate and bleeding rate.

The phenomenon where coarse aggregates sink due to gravity and water arises when the concrete mixture is placed for a long duration is referred to as water bleeding. Large cement particles, unreasonable aggregate gradation, excessive moisture release from aggregates, additives, etc. contribute to water bleeding in concrete. Water bleeding affects the workability and mechanical properties of concrete. Severe water bleeding results in severely deteriorated mechanical and durability performances of concrete as well as concrete deformation and the excessive development of incipient cracks. The water bleeding rates of coral aggregate concrete before and after modification were obtained experimentally, as shown in Figure 12. The water bleeding rate of the concrete increased significantly, which was consistent with the coral aggregate replacement rate. At the same replacement rate, the bleeding rate of the modified coral aggregate concrete was lower than that before the modification. At 0% replacement rate, water was completely used for aggregate pore filling and cement hydration, and no water bleeding occurred. When the replacement rate was 100%, the bleeding rate of the modified coral aggregate concrete decreased by 47.4% compared with that of coral aggregate concrete. This was primarily because the modified aggregate surface formed a dense slurry shell, thus significantly reducing the water absorption and discharge effects of the coral aggregates, and hence a significant improvement in the bleeding rate of the coral concrete. In addition, after the pre-wet treatment of the coral aggregates, a dense cement gel was formed at the aggregate-cement interface during the mixing process, which prevented the internal water escape of the coral aggregates and improved the water bleeding of the coral concrete.

### 3.4. Axial Compressive Strength of Concrete

**Figure 13:**
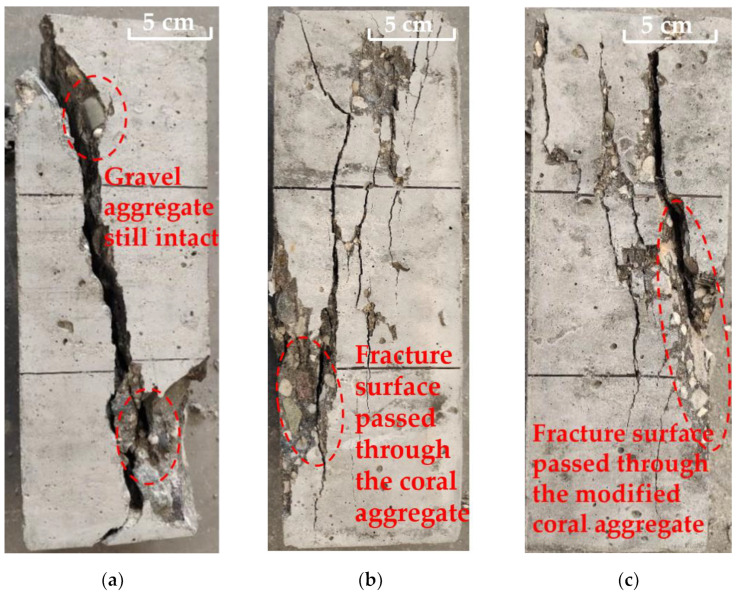
Failure mode of specimens: (**a**) gravel aggregate concrete specimen; (**b**) coral aggregate concrete specimen; (**c**) modified coral aggregate concrete specimen.

**Figure 14:**
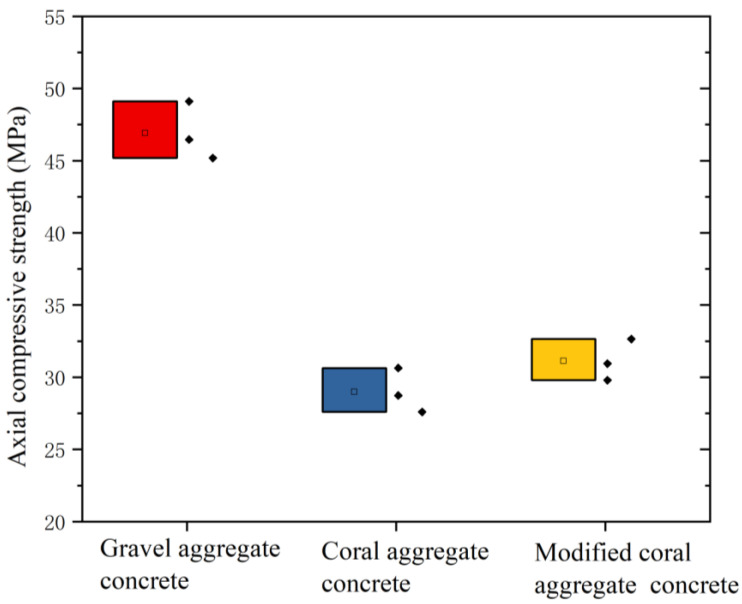
Axial compressive strength of specimens.

Before the compressive strength test, the density of coral aggregate concrete and modified coral aggregate concrete was measured. As shown in Table 7, it can be seen that the density of modified coral aggregate concrete was slightly higher than that of coral aggregate concrete, and the density of both was bigger than that of ceramsite lightweight aggregate concrete. Axial compression tests were performed on gravel aggregate concrete, coral aggregate concrete, and modified coral aggregate concrete test specimens. During the loading process, numerous tiny cracks first appeared near the corners of the specimens. As the load continued to increase, the cracks continued to extend toward the opposite corner, whereas the number, length, and width of the cracks increased. Subsequently, the cracks continued to lengthen and widen, eventually penetrating through the entire specimen; additionally, cracks appeared obliquely along the specimen, as shown in Figure 13. The gravel aggregate concrete, coral aggregate concrete, and modified coral aggregate concrete test specimens indicated damage along the diagonal direction of the specimens. This was due to the frictional force between the upper and lower bottom surfaces of the specimen and the pressure on the specimen caused by the instrument during loading. Friction provides redundant constraints such that the maximum stress of the test specimens is near its diagonal. Despite the diagonal damage pattern of the specimens, more vertical cracks appeared in the coral aggregate concrete specimens. Due to the low strength of coral aggregate, cracks first formed near the coral aggregates. Then the miniscule cracks lengthened and widened with the load increased, thus resulting in more vertical cracks. For the gravel aggregate concrete, cracks appeared in the cement firstly. With the load increased, micro-cracks gradually converged and finally formed a continuous main crack. The axial compressive strength of the specimen was obtained via an axial compression test, as shown in Figure 14. The axial compressive strength of the coral aggregate concrete was much lower than that of the gravel aggregate concrete. The axial compressive strength of the gravel aggregate concrete was 46.92 MPa, whereas that of the coral aggregate concrete was only 28.99 MPa (coral aggregates) and 31.13 MPa (modified coral aggregates). The fracture surface of modified coral aggregate concrete passed through the aggregate. However, the gravel aggregate concrete broke along the interface between aggregate and cement, with the same loading conditions and mix proportion of concrete. Due to the low strength of the modified coral aggregate, the crack was less hindered during expansion. Therefore, the modified coral aggregate concrete was more likely to fail than gravel aggregate. The water absorption and release of the coral aggregates may affect the mechanical properties of the concrete in terms of two aspects. First, the water release of the aggregate will increase the W/C of concrete and degrade its mechanical properties. Second, during concrete hardening, water released from the aggregate is vital to internal curing and thus the concrete strength. Owing to these reasons, the modification of superfine cement does not significantly improve the compressive properties of coral aggregate concrete. To avoid the adverse effects of unstable water absorption and discharge, a precise proportion of concrete, which is difficult to achieve in practical engineering, must be ensured. Therefore, although superfine cement modification cannot significantly improve the strength of concrete, it can considerably diminish the water absorption and discharge of aggregates, thus avoiding the adverse effects of unstable water absorption and desorption.

## 4. Conclusions

In this study, superfine cement was used to modify coral aggregates, and the physical and mechanical properties of modified coral aggregates were experimentally investigated. Subsequently, the effect of superfine cement modification on the workability and strength of coral aggregate concrete was investigated. The main results obtained were as follows:

(1) The water absorption of the modified coral aggregates increased with the slurry W/C. When the W/C was less than 1.25, the variation in the water absorption rate was not apparent; when the W/C exceeded 1.25, the slurry was overly thin, which prevented the formation of a slurry shell on the surface of the coral aggregate; consequently, the water absorption increased significantly with the W/C. After the coral aggregates were modified by superfine cement slurry, the water absorption of the aggregate reduced significantly, and the coral aggregate reached the saturated surface dry state after soaking for 1 h.
(2) The Cr of the modified coral aggregate was higher than that of the unmodified coral aggregate when the curing time was shorter. When the W/C of the cement slurry exceeded 1.25, the Cr of the modified coral aggregate started to be lower than that of the unmodified coral aggregate after 7 d of curing and then decreased significantly as the curing time increased. A slurry with a W/C greater than 1.25 could not be solidified for a long time; hence, a compact slurry protective layer could not be formed on the aggregate surface. The Cr of the aggregates did not change significantly as curing progressed.
(3) As the coral aggregate replacement rate increased, the slump and expansion of the concrete increased; meanwhile, at the same replacement rate, the slump and expansion of the modified coral concrete were significantly lower than those before modification. The slump and expansion of the modified concrete decreased by approximately 16.14% and 14.97% at 100% replacement rate, respectively. The dense slurry shell formed on the surface of the modified aggregate significantly reduced the water absorption and discharge effects of the coral aggregates, and the water secretion rate of the modified concrete was 47.4% lower compared with that before the modification.
(4) After the axial compression failure, the gravel aggregate concrete, coral aggregate concrete, and modified coral aggregate concrete exhibited damage along the diagonal direction of the specimens. Compared with the gravel aggregate concrete, the coral aggregate concrete showed more vertical cracks. The axial compressive strength of the coral aggregate concrete was approximately 60% that of the gravel aggregate concrete. The effect of superfine cement modification on improving the compressive properties of the coral aggregate concrete was insignificant.
(5) Based on the test results, it is suggested that the W/C of superfine cement shall be 1.25. The coral aggregate shall be cured for not less than 7 days after modification, and the soaking time of the modified coral aggregate shall not be less than 1 h.

## Figures and Tables

**Table 1:** Bulk density, apparent density, and porosity of coral aggregate samples.

Physical Properties	Coral Aggregate				
	Sample 1	Sample 2	Sample 3	Average	Standard Deviation
Bulk density (kg/m^3^)	998	1065	1007	1023	36
Apparent density (kg/m^3^)	2437	2509	2481	2475	36
Porosity (%)	59.0	57.6	59.4	58.7	1.0
Crush index (%)	31.9	31.3	32.0	31.7	0.4
Water absorption (%)	12.96	12.88	13.09	12.98	0.11

**Table 2:** Performance index of superfine cement.

Specific Surface Area (m^2^∙kg^−1^)	Particle Size Distribution (μm)			Compressive Strength (MPa)		Flexural Strength (MPa)	
	D50	D90	Average	3 d	28 d	3 d	28 d
800	≤3.5	≤10	3.5	≥40	≥70	≥8	≥11

**Table 3:** Performance index of cement.

Specific Surface Area (m^2^∙kg^−1^)	Standard Consistency (%)	Setting Time (min)		Compressive Strength (MPa)		Flexural Strength (MPa)	
		Initial	Final	3 d	28 d	3 d	28 d
360	28.00	225	295	33.6	55.7	6.3	8.6

**Table 4:** Chemical composition of artificial seawater used in current study.

Chemical	NaCl	MgCl_2_	Na_2_SO_4_	CaCl_2_
Concentration (g/L)	24.53	5.2	4.09	1.16

**Table 5:** Sea sand particle gradation.

Sieve Diameter	4.75 mm	2.36 mm	1.18 mm	600 μm	300 μm
Cumulative percentages of sieve residue (%)	4.0	14.0	29.4	49.0	68.6

**Table 6:** Mix design of concrete (kg/m^3^).

Number	Replacement Rate of Aggregate	Water	Cement	Sea Sand	Aggregates			Water Reducer
					Gravel	Coral	Modified Coral	
P0	0%	200	500	830	1074	0	0	0.05%
P25W	25%	200	500	830	805.5	175.3	0	
P25G		200	500	830	805.5	0	175.3	
P50W	50%	200	500	830	537	350.6	0	
P50G		200	500	830	537	0	350.6	
P75W	75%	200	500	830	268.5	525.8	0	
P75G		200	500	830	268.5	0	525.8	
P100W	100%	200	500	830	0	701.1	0	
P100G		200	500	830	0	0	701.1	

Note: “W” and “G” refer to the coral aggregates before and after modification, respectively.

**Table 7:** Density of different kinds of concrete.

	Coral Aggregate Concrete	Modified Coral Aggregate Concrete	Gravel Aggregate Concrete [59]	Ceramsite Lightweight Aggregate Concrete [60]
Density (kg/m^3^)	2169	2233	2400–2600	≤1950

## Data Availability

Data available on request.
